# Advances of Tumorigenesis, Diagnosis at Early Stage, and Cellular Immunotherapy in Gastrointestinal Malignancies

**DOI:** 10.3389/fonc.2021.666340

**Published:** 2021-08-09

**Authors:** Haipeng Zhu, Xiaojun Liu

**Affiliations:** ^1^Precision and Personalized Cancer Treatment Center, Division of Cancer Diagnosis & Therapy, Ciming Boao International Hospital, Boao Lecheng International Medical Tourism Pilot Zone, Qionghai, China; ^2^Stem Cell and Biotherapy Technology Research Center, Xinxiang Medical College, Xinxiang, China; ^3^Division of Cellular & Biomedical Science, Ciming Boao International Hospital, Boao Lecheng International Medical Tourism Pilot Zone, Qionghai, China

**Keywords:** gastrointestinal cancers, tumor microenvironment, tumorigenesis, early detection, cellular immunotherapy

## Abstract

Globally, in 2018, 4.8 million new patients have a diagnosis of gastrointestinal (GI) cancers, while 3.4 million people died of such disorders. GI malignancies are tightly relevant to 26% of the world-wide cancer incidence and occupies 35% of all cancer-associated deaths. In this article, we principally investigated molecular and cellular mechanisms of tumorigenesis in five major GI cancers occurring at esophagus, stomach, liver, pancreas, and colorectal region that illustrate high morbidity in Eastern and Western countries. Moreover, through this investigation, we not only emphasize importance of the tumor microenvironment in development and treatment of malignant tumors but also identify significance of M2PK, miRNAs, ctDNAs, circRNAs, and CTCs in early detection of GI cancers, as well as systematically evaluate contribution of personalized precision medicine including cellular immunotherapy, new antigen and vaccine therapy, and oncolytic virotherapy in treatment of GI cancers.

## Introduction

Gastrointestinal (GI) malignant tumors generally cover esophagus, stomach, liver, pancreas, as well as colorectal region. Most primary cancers from those parts worldly covered 26% of cancer incidence and 35% of all deaths related to cancer, while 4.8 million new cases were yearly diagnosed and 3.4 million people lost their lives due to those malignancies, statistically in 2018 (1). Currently, clinical scientists are segmentally concentrated on contribution of the tumor microenvironment (TME) to tumorigenesis and resistance to anticancer therapy (2–4), feasibility of precise methodologies in detection of GI cancers at early stage based on tumor growth-associated serological and histochemical changes (5–12), as well as personalized application of biomedical therapies in reduction of tumor size and control of metastases (13–20). More importantly, through combating with cancers over several centuries, many clinicians eventually and principally come back to the nature of malignant tumor, in which an outside exhibition of malignant tumors is uncontrolledly growing but the inside mechanism shows primary inhibition of immune surveillance and cytotoxic activity locally and systematically.

## Tumor Microenvironment and Tumorigenesis in Gastrointestinal Cancers

### Tumor Microenvironment (TME)

Like Achilles, if we cannot lift him from the ground, he will be alive last long. The Achilles malignant tumors that depend on is the TME. The TME is more like a matrix, in which cancer cells communicate with non-transformed cells through a variety of cytokines, chemokines, growth factors, as well as inflammatory mediators, purposely to maintain a dynamic equilibrium in causing metastases and resisting to physical, chemical, and immune pressure (2, 21, 22). The following components principally play critical roles in tumorigenesis and metastasis, including dendrite cells (DCs), T lymphocyte, natural killer cells (NKs), tumor-associated macrophage cells (TAMs), cancer-associated fibroblast cells (CAFs), and myeloid-derived suppressor cells (MDSCs) as well as transform growth factor β (TGFβ).

#### Dendrite Cells (DCs)

DCs, as antigen-processing vehicles, are tightly communicating with tumor-infiltrating T cells in purpose of elimination of carcinogenic cells and prevention of local tumorigenesis and systematical metastases. Primarily, DCs are functional to activate CD4^+^ and CD8^+^ T cell *via* antigen presentation, in which immature DCs intake the antigen to promote DCs maturation (23–25). However, based on hypoxic and inflammatory conditions in the TME, T cells activated by tumor-associated antigens are much less produced due to reduction of DC maturation, accumulation of immature DCs, and specific DC-mediated impairment of T-cell response (26, 27). Thus utilizing immune checkpoint inhibitors (ICIs) not only decrease programmed cell death protein 1 (PD-1) activity on DCs but also increase DC activity promoted *via* amonophosphoryl lipid A produced by E. coli, both of which strongly improve efficiency and efficacy of cellular immunotherapy to GI cancers (28–34).

#### T Lymphocytes

T lymphocytes inside or around the TME are the majorly powerful enforce to fight with cancer cells, in which tumor-infiltrating lymphocytes (TILs) have been purposely used to treat GI cancers and TILs expressed in the TME are closely relevant to therapeutic efficacy (35–40). Generally, cytotoxic CD8^+^-T cells are antigen-processed and have ability to eliminate tumor cells, whereas CD4^+^ T helper 1 cells (Th1) activate cytotoxic T cells to secrete interleukin-2 (IL2), interferon gamma (IFN*γ*), and tumor necrosis factor (41). Thereby, the higher expression of cytotoxic CD8^+^-TIL on biopsy, the better prognosis whether with cancer treatment or not (35–39). However, regulatory CD4^+^ cells, such as Th2 and Th17 group, focus on promoting tumor growth (41). Furthermore, CD4^+^ T regulatory cells (Tregs) with FOXP3 and CD25 expression release IL10, TGF-β, and cytotoxic T-lymphocyte antigen 4 (CTLA4), which tremendously diminish immune surveillance (42, 43). Expression of Treg cells in biopsy represents a negative prognosis (43). Interestingly, in Hodgkin’s lymphoma, Treg cells inhibit malignant cell growing, which show a good prognosis (44–46). More interestingly, γδT cells stained in the TME directly destroy transformed cells and upregulate activity of DCs, cytotoxic T cells, and NK cells in most malignant tumors (47–50). Invertedly, *in situ* production of IL17 *via* γδT cells recruits MDSCs and increases program death protein-ligand 1 (PD-L1) expression that is beneficial for tumorigenesis and metastases (51, 52). Due to contradictory results, whether presentation of γδ TILs in the TME is an eligible biomarker to evaluate prognosis has always been investigated.

#### Natural Killer Cell (NKs)

NK cells, another category of TILs, are characterized with CD16^dim^CD56^bright^ subset mostly in circulation, whereas with CD16^bright^CD56^dim^ subset predominantly in mucosa, in which CD56^bright^ NK cells have cytotoxic action directly on cancer cells independent of antigen presentation (53). Different from functionally killing tumor cells and cancer stem cells (CSCs) in the bloodstream, NK cells show reduced cytotoxicity on transformed cells in the TME with producing less IFN*γ* (54). Unsurprisingly, through combating with immune defense system, malignant cells also develop several tools to dodge NK cell-mediated elimination including activation of inhibitory receptor of NK cells and reduction of NK cell activity induced by platelets and TGFβ (55). However, due to NK-mediated induction of DC cells and increased activation of more NK cells *via* ICI treatment, the existence of NK cells in biopsy highly predicts a good prognosis in GI cancer therapy (54, 56–58).

#### Tumor-Associated Macrophage (TAMs)

Macrophage cells are essentially in regulation of individual immunity through activating adaptive immunity, healing the wound, and kicking off infectious effectors, in which M1 type macrophages with IL12^bright^IL10^dim^ are majorly activated by IFNγ and lipopolysaccharide, whereas M2 type macrophages with IL12^dim^IL10^bright^ are typically activated by glucocorticoid and IL4/13 (59). M1 macrophages produce higher level of IL12 and drive the recruitment of Th1, which promotes antitumor activity, while M2 macrophage-mediated high secretion of IL10 largely sends votes to tumorigenesis. Within the TME, cytokines and inflammatory agents predominantly polarize M1 macrophage to M2 subtype, which produces TAMs (60). There are several receptors commonly expressing TAMs, such as the mannose receptor and scavenger receptor class A, in which blocking scavenger receptor class A *via* monoclonal antibody amplifies cytotoxicity of NK cells to transformed cells in the TME (61). In addition to immunosuppression, TAMs also notably increase tumor angiogenesis, especially at hypoxic area or hypoxia-induced tumor necrosis region where oligonucleotide microarray of aggregated TAMs shows extensive activation of the transcriptional zones encoding angiogenic factors such as vascular endothelial growth factor and endothelin (62). Furthermore, TAMs are strictly relevant with tumor invasion and metastases, and largely prevention of chemotherapy-induced apoptosis in GI cancers (63–65). These discoveries firmly identify high expression of TAMs in biopsy as a poor signal of prognosis in cancer therapy.

#### Myeloid-Derived Suppressor Cells (MDSCs)

MDSCs have two subpopulations including monocytic group and granulocytic group that are characterized through expression of different kinds of membrane markers and immunosuppressive components (66). MDSCs exhibit two directions in promotion of tumorigenesis and metastases including accelerating tumor angiogenesis (67) and inactivating innate and adaptive immune function through producing high concentration of reactive oxygen and nitrogen species (68), slowing down amino acid-mediated T-cell activation and proliferation by less production of arginine (69) and L-cysteine (70) within the TME and reducing intratumoral migration of cytotoxic T cells by peroxynitrite-modified chemoattractant CCL2 (71), as well as enlarging Treg cell-dependent immunosuppression *via* IL10, TGFβ, IFNγ, and CD40-CD40L interactions (72, 73).

Impressively, MDSCs have a triple communication with DCs, NK cells, and TAMs. In the TME, MDSC-TAM complex significantly regulates production of IL10 and IL12, in which IL10-knockdown animal model verifies that MDSC-dependent IL10 level is the key factor of reducing IL12 production through influencing IL12 transcription (74). But cytokine- and inflammatory mediator-induced production of MDSC-TAM complex directly leads to high level of IL10 and low level of IL12 in the TME, which promotes recruitment of Treg cells and predominantly inactivates antitumor function of cytotoxic T cells and NK cells (75). Moreover, higher IL10 production also increases IL4 expression, which basically supports more transformation of TAMs (76). Recent investigation found that granulocytic MDSC-induced inflammation decreased NKG2D expression, an activating receptor of NK cells, which reduced cytotoxicity in the TME (77), whereas monocytic MDSCs amplified expression of activating ligand Rae1 on NK cells to increase killing activity through NKG2D, which subsequently lysed MDSCs (78). Furthermore, combining with ICIs and N803, an IL15 superagonist, NK cell therapy increased cytolysis of MDSCs in squamous cell carcinoma (79). Also, another case in treatment of head and neck squamous cell carcinoma reported that orally bioagent SX-682, a small-molecule inhibitor of CXCR1 and CXCR2, tremendously abolished MDSC aggregation and amplified activity of infiltrating NK cells in the TME (80). These investigations strongly indicate that MDSCs suppress NK cell-mediated elimination of cancer cells in the TME. Through studying effects of MDSCs on DCs in melanoma patients, the evidence showed that MDSCs decreased antigen presentation of DCs, inhibited incubation of immature DCs, and blocked recruitment of DCs to cytotoxic T cells, all of which were activated *via* IL23-mediated Th17 cell pathway (78, 81, 82). More evidences showed that IL23-mediated Th17 cell proliferation damaged adaptive and innate immunity and significantly promoted tumor metastases (78, 83). Therefore, MDSCs primarily enhance TAM-induced immunosuppression and selectively downregulate DC and NK cell activity in promotion of tumor growth and metastases.

#### Cancer-Associated Fibroblast Cells (CAFs)

Healing tissue damage with scar essentially needs participation of fibroblast cells, in which paracrine signals transform residential fibroblasts to myofibroblasts that produce TGFβ and α-smooth muscle actin (84, 85). However, in the TME, myofibroblast cells independent from mutations in cancer cells or the epithelial-mesenchymal transition (EMT) are primarily recognized as CAFs that are abundantly expressed in most malignant tumors (86). In purpose of constantly activating proliferation and metastases, CAFs produce various kinds of growth factors such as hepatocyte growth factor, fibroblast growth factor, and insulin-like growth factor 1 (87). CAF secretion partially activates expression of vascular endothelial growth factor that highly causes angiogenesis (88). Cytokines and chemokines produced by CAFs are functional on cytotoxic T cells, Treg cells, and macrophages, which cause both immune enhancement and immunosuppression (89). Differently, CAF-secreted IL6, CXCL9, and TGFβ reduce T cell response to cancer cells, especially cytotoxic T cells (90), whereas CAF-mediated production of CXCL12 (91) and inactivation of focal adhesion kinase in cancer cells (92) amplify antitumor effects through diminishing stromal fibroblast activation. Moreover, CAFs in the TME also contribute to building up the extracellular matrix (ECM) that consists of fibrovascular cores and remodeling enzymes for increasing tumor density to resist selective pressure (90, 93), while TGFβ-activated CAFs crucially stop activity of anticancer medicine (94, 95). Collectively, CAFs promote tumorigenesis and metastasis through modifying the ECM, utilizing growth factors for angiogenesis, and preventing drug access to reduce therapeutic effects.

#### Transform Growth Factor β (TGFβ)

TGFβ almost regulates all types of human cells and acts as a double agent in the TME (96, 97). Generally, TGFβ secretion is locally responsible to maintain homeostasis, and higher level of TGFβ created by blood platelet and stromal components is responsible for healing the injury and regeneration; however, in the TME, not only stromal cells produce TGFβ through paracrine but also malignant cells autocrinally generate more TGFβ (98). Being a cytokine, TGFβ activates signal transduction through Smad-dependent or Smad-independent pathways, in which Smad-dependent signaling demonstrates that TGFβ-mediated phosphorylation of Smad family protein, Smad2 and Smad3, *via* TβR1 receptor interplays with the cofactor to form the complex that is subsequently translocated into the nucleus and binds with Smad binding element to activate gene transcription (99, 100); however, to Smad-independent signaling, TGFβ activity is amplified mostly through mitogen-activated protein kinase (MAPK) pathway that is activated *via* p38, c-Jun amino terminal kinase, extracellular signal-regulated kinase, and nuclear factor-κB as well as phosphatidylinositol 3 kinase-Akt (PI3K-Akt) (101, 102). The dual roles of TGFβ showing at early and advanced stage of cancers are not due to the change of TGFβ structure, but are mediated through mutant TGFβ receptors as well as interaction of cancer cell-secreted TGFβ with the whole stromal cells such as dendritic cells, T cells, NK cells, TAMs, CAFs, and MDSCs. To suppress tumor growth, TGFβ arrests cell cycle through inhibiting cyclin dependent kinase and c-Myc pathways (103), induces cell apoptosis through activating JNK and Fas pathway, and antagonizing Bcl-2, Bcl-X, and survivin (104), and eventually prevents cell immortalization through regulating reverse transcriptase activity of human telomerase (105). To support tumorigenesis and metastases, paracrinal and autocrinal TGFβ enhances immunosuppression through diminishing immune surveillance maintained by DCs, cytotoxic T cells, and NK cells as well as cytokines such as IL2 and INF*γ* (22), amplifies angiogenesis through activation of ALK1 and ALK5 pathway and more production of vascular endothelial growth factor and connective tissue growth factors in epithelial cells and fibroblasts (97), and induces the EMT for promoting metastases through Smad-dependent and Smad-independent pathway (98), as well as accelerates malignant cells invasiveness through remodeling the ECM and decreasing TβR2 signaling and miRNA-mediated protein regulation (99).

Furthermore, hyperactivation of TGFβ signaling in advanced-stage malignant tumors also swiftly decreases efficacy of ICI therapy in the TME (106), whereas malignant tissue insensitive to ICI treatment exhibits a high expression pattern of TGFβ1 (107). Neutralized monoclonal antibodies individually targeting on TGFβ or dual recognition of TGFβ and PD-1/PDL-1 have achieved significant antitumor effects in treatment of liver cancer, pancreatic cancer, lung cancer, and urethral cancer (108, 109). Therefore, once malignant cells finish the transition from locally seeding to angiogenesis, TGFβ ultimately abandons the antitumor arm and majorly serves to immunosuppression and metastases.

### Tumorigenesis in Gastrointestinal Cancers

#### Esophageal Cancer

Esophageal adenocarcinoma (EAC) and esophageal squamous cell carcinoma (ESCC) are two major malignant exhibitions at esophagus. Epidemiological studies demonstrate that multiple factors cause EAC and ESCC, such as smoking, obesity, gastroesophageal reflex disorder, and Barrett’s esophagus, as well as epithelial injury *via* frequently intaking hot solution, all of which indicate that chronic inflammation is mainly responsible for malignant transition (110). In chronic inflammation-mediated esophageal cancers, not only IL6-STAT3 (signal transducer and activator of transcription 3) pathway increases epithelial proliferation and apoptotic resistance in Barrett’s esophagus and EAC but also neoplastic cells utilize STAT3 signaling to promote tumorigenesis, angiogenesis, and metastases (111, 112). To ESCC, increased production of IL6 causes a poor prognosis following neoadjuvant radiochemotherapy, while higher expression of STAT3 at surgical resection correlates to increased mortality; however, siRNA-activated inhibition of IL6/STAT3 signaling augments chemotherapy sensitivity, accelerates apoptosis, and reduces angiogenesis, as well as decreases the EMT formation for metastases (113, 114).

More importantly, in EAC and ESCC, high expression of CD38-positive MDSCs is abundantly found in the peripheral blood at advanced stage, while Daratumumab binding with CD38 expression reduces tumor growth (115). Previous studies reported that MDSCs increased recruitment of Treg cells in maintenance of immunosuppression in the TME (116). It is highly possible that MDSCs promote malignant invasion through increasing Treg cell activity, in which the higher level of Treg cells, the stronger prediction of tumor invasion, metastasis, disease severity, and reduced overall survival (114, 117). Furthermore, due to the TME in esophageal cancers, especially in ESCC, enormously supporting transition of M1 macrophages to TAMs, infiltrating TAM-based immunosuppression (113), and TAM-mediated upregulation of Treg cell activity tremendously promote the development of EAC and ESCC through STAT3 signaling (118). Additionally, TGFβ directly increases CAF production in the TME, because of which, CAF-based secretion of vascular growth factors accelerates metastases in ESCC, whereas inhibition of CAF activity in EAC notably improves overall survival associated with an excellent prognosis (119). Collectively, interplay of various chronic inflammation conditions activates tumorigenesis in esophageal cancer.

#### Gastric Cancer

Similar to esophageal cancers, most environmental issues are directly and indirectly functional on generating gastric malignancy including high-salt food, pickled food, smoking, nitrates, and food preservers (120). But malignant tumors occurring at cardia and gastroesophageal junction are mostly related to gastroesophageal reflux, intestinal mucosa transition, and chronic mucosal infection caused by H. pylori (Hp) (121). External factors and internal issues confirm the contribution of chronic inflammation in promotion of gastric cancer (122, 123). Two pathological categories in gastric cancer are intestinal type and diffuse type, in which intestinal type is closely related to Hp infection in older males and less morbidity in last several decades worldly, while diffuse type is characterized with loss of E-cadherin expression, frequently occurring in young people unisexually, and less correlating with precancerous lesion (124).

As extensively reported, microorganisms make a great contribution in stimulation of tumorigenesis through dysregulation of cycle progression, protein translation, and cell survival. Epstein Barr Virus (EBV) and Hp are frequently emphasized due to mostly triggering PI3K-Akt pathway and Wnt/β-catenin pathway, respectively (121, 125). Crucially, mutant PI3KAc, an oncogene encoding a catalytic subunit of PI3K_α_, is remarkably expressed in up to 42% EBV-associated gastric adenocarcinoma, which predominantly causes DNA hypermethylation, especially promoting tumor suppressive gene CDKN2A hypermethylation (125). Moreover, amplification of mutant PI3KAc swiftly increases angiogenesis stimulated by VEGF and EGF through TGFβ-CAFs pathway (125). Also blocking oncosuppressive gene, PTEN, activates Akt expression and phosphorylation of Akt, which strongly dysregulates cell apoptosis, tumor invasiveness, and radiosensitivity (126). miRNA-21 targeting on PTEN expression rises proliferation and invasiveness of gastric cancer (126). Dysfunction of Wnt/β-catenin pathway occurs in about 70% gastric cancer patients, in which two virulence factors of Hp, CagA and VacA, are mainly responsible for such dysregulation (127). CagA not only interplays with E-cadherin to increase β-catenin accumulation in cytoplasm and nucleus but also activates CDX1 and P21 genes also firmly responsible for transition of Hp-infected gastric epithelium to mesenchymal stemness such as CSCs (128). Interestingly, chronic inflammation induced *via* Hp infection reprograms SALL4 expression, which directly promotes intestinal metaplasia to neoplastic transformation (129), whereas increased interaction of SALL4 with Wnt/β-catenin signaling also accelerates lymphatic node metastases in gastric cancer patients (130). Furthermore, unsurprisingly, TGFβ is a valid sponsor in upregulation of Hp-induced EMT (131).

Activation of EGFR is responsible for up to 30% gastric cancer cases, in which increased EGFR expression or mutant EGFR is responsible for 5–10% patients and correlates with microsatellite instability molecular subtypes (132). Various coregulators binding with EGFR and TNFα recruit signaling cascades that activate MAPK and PI3K-Akt pathways (133). Nevertheless, HER-2 overexpression exists in 10–30% gastric cancer patients, in which mutant HER-2 occurs in 5% gastric cancer cases, and is more prevalent in 7% microsatellite instability cases and 12% EBV-positive ones (132). Thereby, overexpression of mutant EGFR and HER-2 is highly associated with metastases and poor prognosis in gastric cancer patients (132). Furthermore, specific categories of miRNAs functionally contribute to the development of gastric cancer through increased oncogene expression, reduced expression of oncosuppressive genes, and dysfunction of tumor suppressive proteins; therefore, depending on miRNAs, they are highly likely to become a practically clinical tool in detection of GI cancers and evaluation of prognosis (134).

#### Liver Cancer

Hepatocellular carcinoma (HCC) mostly is diagnosed at the late stage following viral infection, alcohol overconsumption, steatohepatitis, primary biliary cirrhosis, autoimmune hepatitis, and aflatoxin contamination, in which chronic viral infections, such as HBV and HCV, are responsible for 80% cases (135). More researchers believe that oncogenic background changes, dysregulation of up- and downstream molecular pathways, and interaction between stromal cells and cytokines in the TME are tightly connected with cancer cell transition, proliferation, angiogenesis, invasion, and metastasis in HCC.

Single-cell transcriptomic profiling of HCC patients identifies that TME reprogram mainly controls HCC biodiversity, and VEGF-A-mediated regulation of CAFs and TAMs activities is only exhibited in malignant cells, especially in high diversity tumors, but not found in non-malignant hepatocytes (136). This investigation also verifies that individual activation of VEGF-A pathway in HCC is completely responsible to immunosuppression, aggressiveness, and negative prognosis (136). Also, VEGF-A is a critical trigger of angiogenesis in HCC serving for invasion and metastases (137). In HBV- and HCV-mediated hepatitis, chronic infection leads to cirrhotic situation that causes a hypoxic environment, in which hepatocytes upregulate hypoxia-induced factor 1α expression, and consequently that causes overexpression of VEGF at transcriptional level, and finally promotes angiogenesis in HCC (138). Treatment using lenvatinib and sorafenib targeting on VEGFs notably significantly improves prognosis in HCC patients (139). As mentioned previously, TGFβ dysregulated VEGF expression in different kinds of cancers (99); however, TGFβ is a double agent in health and diseased liver. In health liver, Kupffer cells and stellate cells produce most TGFβ except for hepatocytes, while in an injured health liver, temporary activation of TGFβ is functional on nuclear Yes-associated protein and Smad2 phosphorylation (140) that induces the EMT formation dependent on activated hepatic stellate cells and hepatocytes-transformed myofibroblast cells. Moreover, stromal cells in the TME utilize the fibrosis process induced by EMT for metastases, which has been actually verified through miRNA-dependent inactivation of TGFβ pathway in reducing HCC tumorigenesis and improving prognosis (141, 142).

More importantly, overexpression of EGFR and insulin-like growth factor receptors enhances activation of PI3K/Akt/mTOR signaling in HCC, in which atypical PTEN activity upregulates activation of the PI3K/AKT/mTOR pathway (143). Previous researches demonstrated that mutant PTENTs were identified in 5% HCC patients, while reduced or deleted PTEN expression was associated with nearly 50% HCC cases (144, 145). Abnormal expression of PTEN in chronic HBV- and HCV-infected patients constantly stimulates overactivation of PI3K/AKT/mTOR pathway, which constitutively promotes tumor grades and advanced disease stage, and diminishes overall survival in HCC patients (144). Clinically, everolimus and mTOR inhibitor plus a PI3K inhibitor (BKM120), Akt inhibitor (MK-2206), and mTOR/PI3K inhibitor (BEZ235) significantly increase immune cytotoxic activity, especially in treatment of advanced HCC *via* everolimus, in which 40–73% patients quickly catch the disease stable state in a short-time study (146, 147).

Alternatively, in almost 50–70% HCC patients, immunostainings identify overexpression of β-actin in cytoplasm and nucleus especially for tumor cell proliferation and suppression of physiological differentiation; however, under nonmalignant situation, high accumulation of β-actin shows less contribution to HCC formation (148). Furthermore, the early-stage HCC demonstrates that β-actin mutation occurs in 17% HCC patients and these mutant regions cover some specific sites such as phosphorylated site of glycogen synthase kinase-3β (149). Investigation of clustering β-actin resource in HCC shows that mutant β-actins are responsible for 12–26% of patients, whereas mutations in Wnt/β-actin pathway contribute 8–13% cases, both of which predominantly occur in HCV-infected population (149). Clinically, genetic profiling of 603 HCC patients illustrates that either Wnt/β-actin pathway or Myc/Akt pathway is abnormally activated, which is parallel with TGFβ overexpression (150). Interestingly, through activation of Wnt/β-actin pathway, miRNAs regulate TGFβ-mediated ECM formation (151), and more evidences expose that due to the ECM transition, liver has generally become a common site not only for HCC metastasis but also for other solid tumors (152, 153).

#### Pancreatic Cancer

As a deadly disease, pancreatic cancer, like mountain Everest, has always been attractive to many physicians and surgeons in that 5-year survival rate is only 8% (154), in which 90% pancreatic cancer is pathologically defined as pancreatic duct adenocarcinoma (PDAC) and 10-year survival rate is less 4% (155, 156). Principally, in pancreatic cancer, the TME promotes tumor growth and angiogenesis, CSCs focus on anticancer therapy and increased tumor bulk, and interaction between CSCs and the EMT contributes to metastases.

One of pathological characteristics in PDAC is desmoplasia, a state of extensive fibrosis around the primary tumor region, in which the ECM proteins are overexpressed and myofibroblast cells are tremendously identified (157). Clinical evidences also demonstrate that desmoplasia displaying in lymphonodes of the PDAC patients predicts a poor prognosis (157). Desmoplasia occurring in PDAC tightly depends on the TME in that TME-associated secretion of connective tissue growth factors, fibroblast growth factor 2, and TGFβ stimulates desmoplasia formation, especially the absence of anticancer arm of TGFβ caused by reduced Smad4 function depending on RAS-mediated ERK pathway (158). Due to high fibrosis in desmoplasia, the TME exhibits hypovascular and hypoxic conditions, through which pancreatic cancer cells reprogram metabolic pathways, inactivate apoptosis, and amplify proliferation and anticancer resistance, as well as promote invasion and metastasis (159). In purpose of maintenance and enhancement of hypoxia, pancreatic cancer cells produce more angiostatin, endostatin, and pigment epithelium-derived factors, whereas the ECM creates more endostatin, both of which are eventually countering vessel growing (160, 161). Desmoplasia-induced hypoxia, especially *via* hypoxia-induced factor 1α and TGFβ, constantly stimulates production of activated pancreatic stellate cells, in which stellate cells with quiescent vitamin-A contents transform into myofibroblast-like type (162). Through this procedure, normal cellular framework in pancreatic microenvironment steps into the carcinogenic ECM, which is responsible for upregulating secretion of cytokines and chemokines such as matrix metalloproteinases, platelet-derived growth factor, epidermal growth factor, insulin-like growth factor 1, fibroblast growth factor, and stromal-derived factor 1, as well as small leucine-rich proteoglycans and collagen type I, all of which make a great contribution to cancer development and metastasis (162, 163). Moreover, activated pancreatic stellate cells notably participate into immunosuppression in the TME, in which activation of stellate cell-dependent IL6 secretion causes MDSC aggregation deliberately to reduce T-cell proliferation, and increases Treg cell infiltration *via* STAT3 pathway (164). In addition, activated stellate cells also cause more T-cell apoptosis and Th2 cytokine secretion in the TME through upregulating Galectin-1 expression (165). Recently, investigation identified that inhibiting heat-sensitive protein 90 restricted transformation of cancerous pancreatic stellate cells and diminished IL6 production in the TME, which significantly amplified effects of ICIs and increased cytotoxic T cells and Th1 cell infiltration through inactivation of JAK/STAT and MAPK signaling in PDAC (166).

Hypoxia in PDAC not only stimulates production of activated stellate cells but also promotes stemness, in which hypoxic environment indirectly and directly supports CSC creation (167, 168). Growing with pancreatic cancer cells *in vitro* simultaneously, activated stellate cells increase spheroid formation of malignant cells and upregulate expression of stemness markers such as ABCG2, Nestin, and LIN28, which strongly indicates the importance of activated stellate cells in generating CSC niches (169). Specially, TGFβ1 predominantly secreted by activated stellate cells in PDAC stimulates CSC production through negatively regulating L1 cell adhesion molecule expression by TGFβ-Smad2/3 pathway, which causes PDAC to be more progressive (170). More importantly, self-renewal CSCs have become a major barrier of conventional chemoradiotherapy, in which gemcitabine is unable to eradicate CSCs but rather increase the number of CSCs (171, 172). In purpose of maintaining the renewal, increasing the tumor bulk, and promoting metastases, CSCs dysregulate several signal pathways including Wnt/β-catenin, hedgehog, notch, NF-κB, PI3K/Akt, and PTEN (173). Unsurprisingly, CSCs also secret Actin and Nodal that interact with Alk4/7 receptors to form pancreatic cancer cell sphere; however, through reduction of Akl4/7 expression and inhibition of interplay with CSCs, pancreatic cancer cells are more sensitive to gemcitabine treatment and lead to a long overall survival (174).

For entirely understanding the procedure of metastases in PDAC as well as contribution of CSCs in metastases, more investigations evidently show that the EMT insidiously plays a pivotal role during invasion through inducing CSC production continuously (175, 176). The most critical event in forming the EMT is to transform epithelial cells from a normally mature condition into mesenchymal phenotype, which causes morphogenetic changes occurring in embryonic development, organ fibrosis, and tumor metastasis, as well as less expression of epithelial markers such as E-cadherin, desmoplakin, and plakoglobin and overexpression of mesenchymal markers including vimentin, fibronectin, and α-smooth muscle actin (177, 178). Interestingly, hypoxia-inducible factor 1α is functional on expression of SIP1, Snail, Twist1, and Zeb1 indirectly or directly, while hypoxia-inducible factor 2α directly regulates Twist1 expression, both of which enlighten the essential role of hypoxia in induction of the EMT formation (179–181). Impressively, Zeb1 suppresses E-cadherin expression to promote metastases through downregulating expression of stemness inhibitors such as miR-203 and miR-200, in which TGFβ directly causes upregulation of Zeb1 signaling in the EMT (176, 182). Furthermore, circulating tumor cells (CTCs) depending on the EMT-mediated extravascular invasion and migration occupy 100-fold increases of CD24^+^ and CD44^+^ expression that are highly exhibited in pancreatic CSCs associated with therapeutical resistance and negative prognosis (183, 184). That TGFβ predominantly secreted by activated stellate cells strongly enhances CSC production in desmoplasia indicates that interaction between CSCs and the EMT crucially supports metastatic infrastructure and pathological progression in PDAC.

#### Colorectal Cancer

Clinical practice using non-steroid anti-inflammable drugs such as COX-2 antagonists but not aspirin exhibits 40–50% reduction at risk of colorectal cancer (CRC), which strongly demonstrates that chronic inflammation initially promotes tumorigenesis in CRC (185, 186). Further evidences verify that COX-2-mediated prostaglandin E2 production is critically responsible for CRC invasion (187). However, recent researches substantially identified that prostaglandin E2 secreted by CAFs essentially correlates with immunosuppression, angiogenesis, and metastases in CRC, in which upregulation of COX-2 expression on CAFs promotes migration and invasiveness (188), reduction of COX-2-mediated miR-335 expression blocks CAF-induced carcinogenesis through restoring PTEN activity (189), and dysregulation of NK cell function by hepatocellular CAFs depends on prostaglandin E2 and indoleamine 2,3-dioxygenase activity (190). Therefore, transformation of normal fibroblasts to CAFs is a milestone in CRC formation, which purposely remodels the TME through reprogramming secretion of the ECM proteins (191). Moreover, CAFs in CRC strongly amplify immunosuppression in the TME through CAF-mediated enhancement of TAM and MDSC activities (192, 193), CAF-dependent promotion of Treg cell proliferation (194), and CAF-induced PDL-1 overexpression on cancer cells (195), as well as CAF-activated reduction of cytotoxic T cell activity (196). Furthermore, exosomes predominantly secreted by CAFs typically contain miR-21, miR-378e, and miR-143, most of which travel from CAFs to cancer cells in response to increased expression of the EMT markers and stemness to become more aggressive phenotype, which is parallel with results obtained from cancer cells transfected with miR-21, miR-378e, and miR-143 (197). Therefore, miR-21-contained vehicles majorly created by CAFs play an essential role in upregulation of cancer cell-based tissue damaging, especially causing more metastases to liver in CRC (198). Eventually, CAF-dependent exosome secretion is inherently in recruitment of CSCs to resist oxaliplatin or 5-fluorouracil treatment in CRC therapy; however, inhibition of exosome production significantly reduces such kind of behaviors (199). In addition, CAF-induced IL17a secretion also notably promotes self-renewal and invasiveness of CSCs and worthfully to know that chemotherapy of CRC unfortunately upregulates IL17a secretion (200).

## Clinical Approaches in Diagnosis of Gastrointestinal Cancers at Early Stage

Currently, conventional screening cancer tools including plasma-based tumor markers, barium enema, GI endoscopy, and computed tomography have been practiced extensively in asymptomatic population for decades of years in purpose of identifying malignant tumors at early stage, in which GI endoscopy is strongly recommended due to high accuracy of histology; however, invasive nature, unhappier feeling, and noticeable cost have conditionally slabbed this optimal practice for a large-scale of screening tests (201, 202). Most of serum tumor markers generally show less specific in early detection of GI malignancies except for αfetoprotein and CA19-9 with highly predictable value of hepatocellular cancer and pancreatic cancer, respectively (203, 204). According to diversity of cytokines, chemokines, and glycoproteins in the TME, it is necessary to comprehensively apply novel biomarkers for early diagnosis of GI malignancies, which are not independent from traditional cancer-screening tests.

### M2 Pyruvate Kinase (M2PK)

Glutaminolysis and glycolysis almost exist in all kinds of solid tumors in that hypoxia in the TME significantly upregulates glycolysis d in malignant tissues (205, 206). Being a rate-limiting enzyme, M2PK plays a crucial role in regulation of glycolysis, in which tetrameric structure of M2PK exhibits high affinity to phosphoenolpyruvate, whereas a dimeric form, named tumor M2PK with low affinity to phosphoenolpyruvate, is predominantly expressed in malignant tumors (207). A large number of evidences demonstrate that increased expression of tumor M2PK in screening samples especially correlates with GI cancers and also has a pan-sensitive to malignancies in brain, thyroid, lung, breast, ovary, cervix, kidney, bladder, and prostate (205, 208–215).

As a meta-analysis study showed, tumor M2PK as a plasma marker had 62.1% sensitivity and 89% specificity in detection of gastric cancer and esophageal cancer (216). According to different stages of gastric cancer, tumor M2PK has 71% sensitivity to metastatic cases, whereas CA72-4 is 57%, while to the cases without metastases, tumor M2PK has 63% sensitivity, whereas CA72-4 is 25% (11). The study focusing on accuracy of tumor M2PK, CEA, CA19-9, and CA72-4 in diagnosis of GI cancers confirms that tumor M2PK is significantly higher in screening esophageal cancer, gastric cancer, and CRC than other three tumor markers, while in pancreatic cancer, tumor M2PK has a similar predictable rate as CA19-9, but more sensitive than CEA and CA72-4 (6). Furthermore, a comparable meta-analysis study found that screening pancreatic cancer through CA19-9 and M2PK together has 60% sensitivity and 95% specificity (11). More conveniently, testing tumor M2PK in fecal samples is significantly sensitive and reliable than fecal occult blood test (FOBT) due to intermittent bleeding or the absence of bleeding in CRC, in which tumor M2PK shows 68.8 to 91.0% sensitivity and 71.9 to 100% specificity, whereas FOBT has 40% sensitivity only or less (217). Therefore, combining with conventional plasma tumor markers and periodic endoscopy examination, tumor M2PK is practically selected as an earlier alert signal in detection of GI malignancies, especially in screening CRC.

### Circulating Tumor Cell and Circulating Tumor DNA

CTCs are tumor cells that have broken away from primary tumors or metastatic sites, and then enter into the blood circulation in purpose of distal metastases (218). Comparing with other peripheral blood cells, the number of CTCs is extremely low, generally 10^-7^ to 10^-8^, and lifetime mostly lasts for several hours (219–221). Currently, using CTCs to screen GI cancers in normal population is not actually realistic due to remarkably low fraction of CTC-positive patients, screening markers less specific for CTCs assessment, and tremendously lower level of CTCs detected at early-stage tumors (222–224). A cohort study of 138 CRC patients at stage I to III demonstrates that postoperative CTC-positive patients who were negative at preoperative CTC test independently predict a really poor prognosis, whereas preoperative CTC-positive patients have similar results as preoperative CTC-negative group in evaluation of prognosis (225). Therefore, CTC test may be highly valid to patients with extensive metastases, post-surgery relapse, or the high-risk population with family history of GI cancers as well as a good candidate in prediction of prognosis after chemoradiotherapy.

Circulating tumor DNA (ctDNA) is a kind of cell-free DNA originally traveling from primary cancer site, metastatic location, or CTCs to the bloodstream, in which the size of ctDNAs is generally shorter than normal cell-free DNAs, lifetime lasts for 16–150 min, and can be released into exosomes (226, 227). Different from CTC test, ctDNAs screening has employed tumor-specific genetic markers and epigenetic markers in discrimination of ctDNAs from normal cell-free DNAs (228), and sensitivity of ctDNA detection has exhibited microscale level through molecular barcoding techniques (229). Furthermore, identification of ctDNAs through low-coverage genome sequencing has not required genomic information of primary malignancy (230), and ctDNA detection using plasma samples is more accurate than in serum due to lower level of wild-type DNA released (231). Through combinative application of both ctDNAs and CTCs techniques as well as through detection of hypomethylation or hypermethylation of ctDNAs at promoter region of cancerous and oncosuppressive genes, ctDNAs have been extensively used in early diagnosis of GI cancers. However, due to few amounts of ctDNAs released into the circulation, ctDNA technique is encountering some challenges in detection of ultra-early stage of GI malignancies (232, 233).

In esophageal cancer, ctDNAs targeting CASZ1, CDH13, and ING2 genes show significant high level of hypermethylation in ESCC patients but mild level in healthy controls, whereas dysregulation of 5-hydroxymethylcytosine expression on ctDNAs also has remarkable sensitivity in screening ESCC; therefore, ctDNA detection based on these two different patterns is highly likely to identify the early-stage esophageal cancer (234, 235). Through screening methylation and hypermethylation of 14 genes in 1193 samples, ctDNA approach has 65% sensitivity and 95% specificity in diagnosis of gastric cancer, whereas in comparison with CEA, CA72-4, CA19-9, and CA50, the higher concentration of ctDNAs tested by *alu*-dependent branch DNA assessment, the more accuracy in early diagnosis of gastric cancer, which has 79% sensitivity and 91.8% specificity (236, 237). Therefore, combining hypermethylation of CASZ1, CDH13, and ING2 genes with increased ctDNA burden is a valuable approach for a large-scale screening of upper digestive cancers. Using somatic copy number aberration (SCNA) *via* low-depth whole genome sequencing technique demonstrates that increased ctDNA burden has area under curve (AUC) value of 0.874 in early diagnosis of HCC and 0.933 at advanced stage of HCC, whereas application of ctDNA panel focusing on eight targeted genes has 83.3% sensitivity and 90.5% specificity in screening HCC; thereby, screening people through a panel containing multiple ctDNAs with increased ctDNA burden comprehensively provides a specific method in diagnosis of HCC (238, 239). To detect pancreatic cancer, several investigations clarify that single ctDNA-mediated tests are not sensitive at the early-stage; however, through combining with conventional serum tumor markers, specific proteins expressed in the TME and methylation at protomer regions of cancer-related genes, ctDNA technique significantly increases sensitivity and specificity in diagnosis of pancreatic cancer (240, 241). Using ctDNAs to detect methylation expression at promoter regions of 17 genes exhibits 91.2% sensitivity and 90.8% specificity to discriminate PDAC patients from chronic pancreatitis group (242). Through examining methylation at both ADAMTS1 and BNC1 genes in screening PDAC, ctDNAs tests remarkably accomplish the accuracy with 94.8% sensitivity and 91.6% specificity (243). Moreover, detection of mutant KRAS through ctDNAs combined with other four protein markers notably has 64% sensitivity and 99.5% specificity in diagnosis of PDAC patients from healthy controls (244). Thereby, the methodology of integrating ctDNAs with KRAS mutation, methylation on targeted genes, and high expression level of tumor-specific proteins predominantly occupies a position in early diagnosis of pancreatic cancers. Similarly to detect early gastric cancer, increased ctDNA burden through *Alu*83 and *Alu*244 is also used to detect CRC, combination of which with methylation of specific gene achieved higher sensitivity in CRC diagnosis (245). Furthermore, using ctDNAs to examine B4GALT1 gene hypermethylation performs 50% sensitivity and 100% specificity in early diagnosis of CRC, whereas hypomethylation of LINE-1 gene tested by ctDNAs yields 65.8% sensitivity and 90.0% specificity in screening CRC (246). Therefore, testing M2PK-positive samples depending on increased ctDNA burden, hypermethylated B4GALT1 gene, and hypomethylated LINE-1 gene is a reliable methodology in early detection of CRC.

### Circulating microRNA

microRNAs (miRNAs) generally composing of 19–24 nucleotides are functional on 3’-UTR of messenger RNA, which effectively regulates targeted protein expression (247). miRNAs are endurable in various physiological changes and resist to RNase activity, thereby circulating miRNAs extensively and stably present in 12 kinds of cell-free body fluids and excretions including serum, plasma, urine, and feces (248). Since downregulation of miR-15 and miR-16 expression was firstly recognized as a novel biomarker in diagnosis of B-cell chronic lymphocytic leukemia (249), clinical professionals have applied circulating miRNAs in detection of GI cancers considerably and identified several unique expression patterns in early diagnosis of esophageal cancer (250, 251), gastric cancer (252), liver cancer (253), pancreatic cancer (254), and CRC (255).

Comparing to healthy controls, the expression panel containing miR-16-5p, miR-197-5p, miR451a, and miR-92a-3p is specially associated with ESCC, whereas the group of miR-16-5p, miR-320c, miR-638, and miR-92a-3p is significantly higher in squamous dysplasia, a precancer pathology, especially miRNA-21 overexpression highly correlating with alcohol-induced ESCC (250, 251). In a cost-effective screening of gastric cancer, 12-miRNA panel shows 87% sensitivity and 68.4% specificity, which is remarkably higher than CEA and CA-19-9 (252). A meta-analysis study of circulating miRNA-mediated detection of HCC demonstrates that the higher expression group of circulating miR-21, miR-122, and miR-223 is more specific in diagnosis of HCC than in healthy, hepatitis, or cirrhosis group, in which confident rates of miR-21, miR-122, and miR-223 are 0.9293, 0.8128, and 0.8597, respectively, especially miR-21 expression in high priority (256). Screening of pancreatic cancer proves that the expression group containing miR-125a-3p, miR-5100, and miR-642b-3p achieves the most promising result in discrimination of cancer patients from healthy ones, which shows 98% sensitivity and 97% specificity (254). Moreover, in diagnosis of CRC, the expression pattern consisting of miR-15b, miR-17, miR-21, miR-26b, and miR-145 has the best predictable performance, especially miR-21 and miR-26b with maximal specificity (257). Recently, circulating exosomes were also considered as the early biomarkers in diagnosis of GI cancers; however, the reliability of this approach mainly depends on analysis of contents within the circulating exosomes, especially miRNAs and cell-free DNAs (258).

Finally, through reviewing 42 investigations significantly relevant to the diagnostic performance of circulating miRNAs in GI cancers, this meta-analysis study concludes that comparing to CEA and CA19-9, circulating miRNAs have become reliable biomarkers in early diagnosis, moderately with 75% sensitivity and 81% specificity, and multiple-miRNA screening assay significantly achieves more accurate result than a single-miRNA test, as well as plasma-dependent miRNAs assay is precisely used in diagnosis of gastric cancer, while serum-based miRNA test is more suitable for CRC detection (259).

### Circular RNA

Circular RNAs (circRNA), non-coding RNA with closed circular form, have been discovered in mammalian cells for decades and structurally characterized through high-throughput sequencing over 10 years, in which circRNAs are functional on the axle of circRNA-miRNA-mRNA responsible for target gene expression, interplay with RNA-binding protein in regulation of target protein activities, and act as posttranscriptional regulators influencing parental gene transcription and splicing (260). Due to strongly resisting to exoribonuclease, circulating circRNAs are stably and extensively expressed in exosomes, serum, plasma, saliva, and urine (261). Since ciRS-7 firstly exhibited sponging action on miRNA-7 to promote carcinogenesis, different kinds of cancers have shown unique existing patterns of circRNAs that are practically applied in diagnosis of malignancy at early stage (261). Moreover, recent investigation demonstrated that artificial expression of circRNA targeting on miRNA21 eliminated miRNA-21-mediated promotion of gastric cancer (262). Therefore, examining expression pattern of circRNAs sponging on cancer-specific miRNAs is highly likely to become a more efficient methodology in early diagnosis of GI malignancies.

Using a pool of circRNAs to screen ESCC identifies that upregulation of circ-DLG1 and circ-TTC17 combining with downregulation of hsa_circ_0001946, hsa_circ_0062459 and circ-SMAD7 has 79% sensitivity and 85% specificity, in which positive samples in this pool test have 5-fold higher possibility in transition to ESCC than normal controls (263). Through analyzing 343 plasma circRNAs expressed in gastric cancer patients and healthy controls, downregulation of hsa_circ_0001017 and hsa_circ_0061276 has the best diagnostic performance with 95.5% sensitivity and 95.7% specificity (264). Another study shows that lower expression of plasma hsa_circ_0000181 has 99.0% sensitivity and 85.2% specificity in gastric cancer (265). Therefore, the expression group of hsa_circ_0001017 and hsa_circ_0061276 and hsa_circ_0000181 is practically becoming a novel biomarker for early diagnosis of gastric cancer. For HCC detection, upregulation of seven circRNAs expression plus downregulation of five circRNAs expression in serum shows the best combinative performance, in which the expression group covering hsa_circ_0004001, hsa_circ_0004123, and hsa_circ_0075792 has 90.5% sensitivity and 78.1% specificity (266). Furthermore, two independent studies identically discover that plasma circ-LDLRAD3 overexpression significantly correlates with pancreatic cancer, in which together with CA19-9, combinative test has 80.3% sensitivity and 93.6% specificity in early diagnosis of pancreatic cancer (267, 268). Thus, integrating conventional tumor markers with circRNA expression highly possibly obtains more precise diagnosis in screening of pancreatic cancer. Similar methodologies are also used in early diagnosis of CRC, in which combining with CEA and CA19-9, the group of downregulated expression of plasma circ-CCDC66, circ-ABCC1, and circ-STIL increases AUC value 0.780–0.855 (269). Another investigation focusing on early diagnosis of CRC shows that using upregulated expression group of hsa_circ_0001900, hsa_circ_0001178, and hsa_circ_0005927 precisely identifies CRC patients in the CEA-negative group with AUC value 0.859 (270). Therefore, combinative patterns of using circRNAs with CEA and M2PK will maximally increase the diagnostic accuracy in screening CRC.

## Cellular Immunotherapy in Treatment of Gastrointestinal Cancers

Cellular immunotherapy is a combinative methodology of applying cellular therapy and immunotherapy in personalized cancer treatment (271–273), in which personalized analysis to an individual cancer patient is the key strategy to achieve a clinically effective treatment (273). Personalized investigation in cancer therapy includes genomic DNA sequencing, genomic exons sequencing, cancer-specific mutant genes sequencing, dysfunction of signaling proteins analysis, neoantigen detection, loss of immune cytotoxicity, and diversity of the TME (274). However, following nearly one century training, oncological clinicians have been used to dogmatically applying conventional chemotherapy and radiotherapy in almost all cancer treatment. Whether therapy-induced immune alterations are responsible for an effective therapy is less attractive in main menu despite the critical role of cellular immunotherapy to malignancy having been successfully exhibited through Dr Coley’s vaccine in 1893 (275). Actually, Coley’s vaccine strongly indicates that turning cold tumor to hot through immune modulation is an essential conversion from treatable to clinically curable. Currently, cellular immunotherapy includes neoantigens and vaccines, adoptive cell therapy, CAR-T/NK techniques, and oncolytic virus (OV) in purpose to recover immune normalization, reduce immunosuppression in the TME, increase tumor-specific antigen expression, and locally rebuild inflammatory conditions, as well as rearrange tumor vasculature.

### Combination of Conventional Chemotherapy and Radiotherapy With Cellular Immunotherapy

For many years, a number of investigations have demonstrated that using pan-cytotoxic chemotherapy in cancer treatment causes a direct immunosuppression (276, 277), PDL-1 overexpression (278), recruitment of CSC-mediated drug resistance (200), and increased VEGFR-1-activated metastases (279), as well as myelosuppression (280), all of which objectively and comprehensively indicate that conventional chemotherapy is not an approach of precise medicine in cancer treatment. Surprisingly, through combining with certain kinds of chemotherapeutic agents, cellular immunotherapy significantly increases overall survival than singly using chemotherapy (281, 282). Further analysis elucidates that chemotherapeutics specifically acting on MDSCs and CSCs to reduce immunosuppression in the TME remarkably amplify efficiency and efficacy of ICI treatment and adoptive cell therapy (282, 283). Previously, clinical professionals supposed that apoptotic cancer cells or chemotherapy-induced mutation should have expressed more neoantigens; however, this idea is highly likely a misconception (284), but through applying histone deacetylase inhibitors or radioactive treatments, neoantigens expressed on cancer cells are notably beneficial for cellular immunotherapy due to enormously increased antigenicity (285, 286). Moreover, boron-neutron capture therapy (BNCT) not only precisely and largely eliminates tumor volume but also produces a large amount of various neoantigens due to radiation-damaged DNAs extensively existing in survived cancer cells, which are specifically utilized by cellular immunotherapy for constantly attacking (287, 288).

### Adoptive Cell Therapy

Adoptive cell therapy includes TILs, cytokine-induced killer cells (CIK), dendritic cell-activated cytokine-induced killer cells (DC-CIK), NK cells, CAR-NK (chimeric antigen receptor-natural killer cells), and CAR-T (chimeric antigen receptor-T cells), in which TIL methodology showed clinical effects firstly on patients with metastatic melanoma in 1983 (289), and currently, TIL (290), DC-CIK (291), NK (292), CAR-NK (293), and CAR-T (294) all have achieved convincing results in cancer treatment, especially through DC-CIK, NK, and CAR-NK approaches.

As given knowledge, DCs are the most powerful antigen presentation immunocytes, which directly command cytotoxic T lymphocytes to attack cancer cells; therefore, depending on DC-mediated recruitment of CIKs through tumor-associated antigens, DC-CIK therapy efficiently and specifically eliminates malignant cells (295). Through three meta-analysis studies of more than 8 thousand patients with gastric cancer and CRC, comparing to individual application of chemotherapy, CIK/DC-CIK plus chemotherapy significantly increases overall survival and progression-free survival in drug-resistant patients, and swiftly improves most adverse events caused by chemotherapy (28, 296, 297). Similar results are also exhibited in CIK/DC-CIK therapy of pancreatic cancer and HCC, especially in advanced-stage patients (298, 299). Interestingly, infusion of allogeneic CIKs has also demonstrated encouraging effects on hematological malignancies (300). Comparing to DC-CIK therapy, as innate immune system, NK cell treatment occupies a unique advantage, in which tumor-associated antigen presentation is not compulsory for NK cell cytotoxicity, cytokine release syndrome is less possible to occur, and human leukocyte antigen matching is not stringently required for donor NK cell infusion; therefore, despite autologous NK cells showing very limited clinical efficacy (301), allogeneic NK cells including semi-allogeneic NK cells, NK cells isolated from umbilical cord blood, and iPSC-derived NK cells all show extraordinary immune killing specificity to malignant tumors without graft-versus-host disease (53, 302). Multiple infusions of allogeneic NK cells to stage-III/IV pancreatic cancer patients significantly increase overall survival and progression-free survival to 13.6 months and 9.9 months, respectively (303), whereas to unresectable primary HCC, are 23.2 months and 15.1 months, respectively (304), both of which clinically identify allogeneic NK cell therapy as a novel and promising methodology in treatment of GI cancers. Recent investigations discovered that PD-1 and PDL-1 expressed on DCs and NK cells reduced DC-activated maturation of cytotoxic T lymphocytes (305, 306) and NK-mediated antigen presentation of DCs (307–312), but through applying ICIs, both DC-CIK and NK therapies exhibit the higher level of cytotoxic activity to cancer cells (313–315).

Through studying the principle of TILs, clinical scientists naively consider that arming T cells from cancer patients with Chimeric tumor-specific Antigen Receptors (CAR-T) is efficient to destroy cancer cells precisely, and CAR-T technique indeed achieves the success in treatment of some kinds of hematological malignancies (316). However, different from attacking an individual malignant cell with unique antigen expression in the bloodstream or in the lymphatic nodes, CAR-T therapy to solid tumors is like using the sharpness of a knife to fight with the thickness of a tank, disadvantages of which include immunosuppression of the TME in solid tumors always primarily focusing on T cells, loss of tumor-associated antigens at the surface of mature solid tumors, and high cell density of inside solid tumors stopping CAR-Ts entering into the bulk, as well as extremely lethal side effects caused by immunotoxicity to the normal cells co-expressing tumor-associated antigens (317, 318). Also, due to continuously selective pressure caused by specific CAR, cancer cells may highly possibly abandon unique antigen expression through endocytosis. Therefore, in purpose of promoting immune normalization but not enhancement (319), rebuilding normal immunity in cancer patients through cellular immunotherapy is far more essential than CAR expression. Due to specific cytotoxicity to malignant tumors and much less possibility of inducing cytokine storm, clinical scientists design CAR-NK technique through equipping NK cells with chimeric antigen receptors, and have applied CAR-NK in patients with relapsed and refractory acute myeloid leukemia to test safety and tolerance (320). Whether CAR-NK therapy has more therapeutical advantages in solid tumors than combinative treatment of DC-CIK plus allogeneic NK cells, clinicians still have a long way to go. Following identification of specific biomarkers of TAMs, MDSCs, CAFs and CSCs, CAR-NK therapy certainly makes a great contribution in breaking through immunosuppression in the TME and strongly provide a beneficiary immune infrastructure for other kinds of cancer therapies.

### Vaccine and Oncolytic Virus

Vaccines used in cancer therapy mainly depend on transferring tumor-associated antigens to antigen presenting cells such as DCs, and then inducing CD4^+^ helper T cells and CD8^+^ cytotoxic T cells to eradicate cancer cells (321). Genetic vaccines including DNA and RNA vaccines are theoretically able to synthesize all sequences encoding targeted antigens; however, according to various levels of protein translation, immunogenicity produced by genetic vaccines is hard to control (322). Despite DNA vaccines achieving pathological regression in treatment of intraepithelial neoplasia caused by human papillomavirus infection (323), clinical trials have not shown effective results in solid tumors therapy such as breast cancer (324), CRC (325), prostate cancer (326), and melanoma (327). Similar to DNA vaccines, messenger RNA vaccines containing 20 epitopes inoculated for patients with advanced-stage GI cancers have not obtained clinical efficacy, although they are safe and have activated immune response to neoepitopes (328). Actually, DC vaccines are more appropriately applied for cancer therapy in that they are *ex vivo* induced through the whole cancer cells, tumor lysates, peptides, DNAs, and RANs, which are strongly functional on tumor-specific cytotoxic T cells to eliminate cancer cells. Therapeutic DC vaccines have been extensively used in treatment of GI cancers at phase II or III trials, in which DC vaccine combining with MAGE peptide safely promotes immune response to tumor-specific antigens and significantly reduces tumor marker expression in nearly 90% advanced GI cancer patients (329), but for accomplishment of more optimal prognosis, prior to using DC vaccines, it is critical to examine NK cell activity in the candidates (330). Furthermore, *in situ* vaccines generated through chemoradiotherapy show more beneficial to cancer patients than conventional vaccines in that there is no screening requisition of positive antigen expression (331, 332). Almost all clinical trials demonstrate that adding ICIs into vaccine therapy remarkably increase vaccine-mediated cytotoxicity, which partially explains why individually using vaccine therapy only achieves suboptimal impact in cancer treatment (333, 334). Currently, the challenges vaccine therapy needs to deal with are multiple immunogenic antigen expression, construction of highly potent vaccine vectors, and breakthrough of immunosuppression in the TME.

Oncolytic virotherapy includes various kinds of viruses targeting a large range of malignant tumors, in which cancer cells are vulnerable to viral infection due to absence of type I interferon system and mutations promoting attachment of viruses (335, 336). In cancer therapy, using adenoviruses as a platform to express tumor-specific antigens and tumor-suppressive proteins has exhibited the promising results in preliminary animal studies, and currently in human studies, telomelysin-targeted adenovirus is testing in treatment of esophageal cancer, whereas evaluation of ideal viral vectors is processing in gastric cancer therapy, as well as more clinical trials are manipulating in treatment of pancreatic cancer, primary HCC, and CRC (337). Currently, clinical scientists are also concentrated on myxoma virus due to absence of infection to human beings and extremely sensitive to cancer cells; however, there still have a distance for clinical application (338). Furthermore, in diagnosis of GI cancers, depending on specific expression of fluorescence and bioluminescence on infected cancer cells, OVs, being tracing signals, are used to accurately expose the border of malignant tissue in surgery, and precisely identify the primary tumor and metastatic microtumors through nuclear medical imaging techniques such as CT, MRI, SPECT, and PET-CT (339–343). Through practicing oncolytic virotherapy, some barriers clinicians have to quell include tumor heterogeneity, general immune elimination of OVs, and capability to hack into tumor bulk and induce more activated cytotoxic T cells in the TME.

## Conclusion

To successfully apply personalized therapy in cancer treatment, it is crucial to set up an assessment of tumor heterogeneity (344). In this review, we seemly underrate the importance of tumoral heterogeneity, in fact, which has been broadly discussed in parts of diagnosis and cellular therapy. Due to high variability of single subclone- or multiple subclone-induced tumor evolution in the same tumor or metastatic regions of the same tumor, as well as in different types of GI cancers, intratumoral genomic heterogeneity is highly beneficial for early diagnosis *via* liquid biopsy (345–347), while intertumoral heterogeneity-mediated changes of temporal and spatial plasticity and stemness in the TME precisely support the significance of comprehensive therapeutic principle (348, 349). Complexity of tumor heterogeneity essentially reflects adaption of genomic heterogeneity to selective pressure and utility of angiogenesis and immunosuppression through the TME for distant metastasis (350–352). Tumor heterogeneity also strongly indicates that rebuilding immune normalization to block interaction of intertumoral heterogeneity with the TME is a practical methodology to alter cancer therapy to chronic disease management.

Through systematically discussing key contributions of various components in the TME to tumorigenesis and metastases, reliability of early diagnostic methodologies, and personalized application of cellular immunotherapy in GI malignancies, it has become more apparent that immunosuppression induced by TAMs, MDSCs, CAFs, and CSCs in the TME and autocrine-paracrine network supported by stromal cells and cytokines and growth factors, especially TGFβ, are critically responsible for resistance to anticancer therapy and significant reduction of overall survival. Therefore, based on personalized investigation of each cancer patient including seeking mutant target genes and neoantigens, evaluating immune cytotoxicity in the TME, and recovering immune normalization, precise cellular immunotherapy is highly likely to change cancer treatment into a state of managing chronic disorders and eventually achieve the coexistence *via* normalization of immune surveillance. Due to complex of the TME, precise methodologies used to detect GI cancers at early stage primarily depend on the personalized combination of M2PK, miRNAs, ctDNAs, cancer-produced exosomes, and CTCs with conventional tumor markers and serologically biochemical examinations, in which CTC methodology is far more beneficial in evaluation of treatment efficacy and prognosis. Prior to treatment, a solid tumor is like an onion, in which anticancer mechanism of each layer is highly changeable, thereby, one step-by-step comprehensive treatment plan primarily includes that using BNCT technique efficiently reduces tumor volume and tears the tumor shell, and then applying OVs targeting TAMs, MDSCs, and CSCs with ICIs or dual recognition antibody of TGFβ and PD-1/PDL-1 maximally destroys immunosuppression and amplifies immunotoxicity of infiltrating T cells in the TME; furthermore, alternative infusion of DC-CIKs and allogeneic NK cells significantly eliminates most cancer cells with or without neoantigen expression. Such a therapy plan remarkably shrinks and eradicates the whole onion layer by layer. For CTCs or temporary formation of metastatic microtumors, CAR-T and CAR-NK therapies predominantly contribute to remove them. Surely, through recovering immune normalization in cancer patients and high-risk population, prevention of cancers in the community is far more critical than diagnosis and treatment. Whether precisely selecting the personalized cellular immunotherapy based on the diversity of the TME or rigidly relying on the unspecific chemotherapy, your choice.

## Author Contributions

HZ made outline, wrote and revised the draft, and finalized the review. XL made outline. All authors contributed to the article and approved the submitted version.

## Conflict of Interest

The authors declare that the research was conducted in the absence of any commercial or financial relationships that could be construed as a potential conflict of interest.

## Publisher’s Note

All claims expressed in this article are solely those of the authors and do not necessarily represent those of their affiliated organizations, or those of the publisher, the editors and the reviewers. Any product that may be evaluated in this article, or claim that may be made by its manufacturer, is not guaranteed or endorsed by the publisher.

## Abbreviation

GI, gastrointestinal; TME, tumor microenvironment; DCs, dendrite cells; NKs, natural killer cells; TAMs, tumor-associated macrophage cells; CAFs, cancer-associated fibroblast cells; MDSCs, myeloid-derived suppressor cells; TGFβ, transform growth factor β; PD-1, programmed cell death protein 1; PD-L1, program death protein ligand 1; ICIs, immune checkpoint inhibitors; TIL, tumor-infiltrating lymphocytes; IL2, interleukin-2; IFN*γ*, interferon gamma; Tregs, T regulatory cells; CTLA4, cytotoxic T-lymphocyte antigen 4; CSCs, cancer stem cells; EMT, epithelial-mesenchymal transition; ECM, extracellular matrix; MAPK, mitogen-activated protein kinase; PI3K-Akt, phosphatidylinositol 3 kinase-Akt; STAT3, Signal Transducer and Activator of Transcription 3; EAC, Esophageal adenocarcinoma; ESCC, Esophageal squamous cell carcinoma; Hp, H. pylori; EBV, Epstein Barr Virus; HCC, hepatocellular carcinoma; PDAC, pancreatic duct adenocarcinoma; CRC, colorectal cancer; M2PK, M2 pyruvate kinase; FOBT, fecal occult blood test; CTCs, Circulating tumor cells; AUC, area under curve; ctDNAs, circulating tumor DNAs; miRNAs, microRNAs; circRNA, circular RNAs; BNCT, boron-neutron capture therapy; CIK, cytokine-induced killer cells; DC-CIK, dendritic cell-activated cytokine-induced killer cells; CAR-NK, chimeric antigen receptor-natural killer cells; and CAR-T, chimeric antigen receptor-T cells; OV, oncolytic virus.
